# Knowledge of Health Care Professionals and Medical Students Regarding Covid-19 in a Tertiary Care Hospital in Nepal

**DOI:** 10.31729/jnma.4995

**Published:** 2020-07-31

**Authors:** Harish Chandra Neupane, Niki Shrestha, Shital Adhikari, Siddeshwar Angadi, Bishow Kumar Shrestha, Basanta Gauli

**Affiliations:** 1 Department of Surgery, Chitwan Medical College, Bharatpur, Chitwan, Nepal; 2 Department of Community Medicine, Chitwan Medical College, Bharatpur, Chitwan, Nepal; 3 Department of Medicine, Chitwan Medical College, Bharatpur, Chitwan, Nepal; 4 School of Nursing, Chitwan Medical College, Bharatpur, Chitwan, Nepal; 5 Department of Anaesthesiology & Critical Care Medicine, Chitwan Medical College, Bharatpur, Nepal

**Keywords:** *COVID-19*, *health care professionals*, *knowledge*

## Abstract

**Introduction:**

The lack of knowledge among health care professionals leads to diagnostic delays, further spread of disease, and poor infection control practices. Health care professionals must be updated knowledge regarding COVID-19. This study aims to assess the knowledge of health care professionals regarding COVID-19 in a medical college in Chitwan.

**Methods:**

A Knowledge, Attitude and Practice Study was carried out in a tertiary care hospital in Chitwan, Nepal from April 22, 2020, to April 28, 2020. The institutional review committee of Chitwan Medical College provided ethical approval for the research. Data were collected with an online questionnaire using Google forms. The questionnaire was sent out to 724 potential responders who included health care professionals from medical, dental, nursing, and allied health sciences in Chitwan Medical College. A convenient sampling method was used for data collection. Data were analyzed using Statistical Package of Social Sciences.

**Results:**

A total of 181 respondents completed the web survey. Overall, a total of 35 (19.3%) respondents were found to have "Good" knowledge; 105 (58%) respondents had "Fair" knowledge and 41 (22.7%) respondents had "Poor" knowledge regarding various aspects of COVID-19. There was no significant difference among the various health professional groups in their knowledge scores under the four knowledge domains.

**Conclusions:**

The study of knowledge of health care professionals could act as a reference for the prevention and better management of COVID-19. This study shows that there is a need to implement periodic educational interventions and training programs on infection control practices for COVID-19 across all healthcare professions.

## INTRODUCTION

The COVID-19 pandemic is exacting a huge toll throughout the world. The capacity of COVID-19 for explosive spread has challenged even the most robust health systems.^1^ Overall, 20% of cases are severe or critical, with a crude clinical case fatality rate currently of over 3%, which is found to increase in older age groups and those with certain underlying conditions.^1^

COVID-19 poses a serious occupational health risk to health care professionals.^2^ The protection of health care professionals and the prevention of intra-hospital infection transmission are vital elements in pandemic response. It is therefore imperative that health care professionals must have updated knowledge regarding COVID-19.^3^ As the literature suggests, the lack of knowledge among HCPs leads to diagnostic delays, further spread of disease, and poor infection control practices.^4^

This study aims to assess the knowledge of health care professionals and medical students regarding COVID-19 in a medical college in Chitwan.

## METHODS

A KAP Study was carried out at a tertiary care hospital in Chitwan, Nepal. The period of the survey was from April 22, 2020, to April 28, 2020. The CMC-IRC reviewed and approved the study-related documents and provided the ethical clearance for the research (Ref: CMC-IRC/076/077-104).

The main instrument to collect data was an online questionnaire using Google forms. The questionnaire was prepared with reference from various current interim guidelines and information for healthcare personnel provided by WHO.^5^ The developed draft survey instrument was distributed to ten randomly selected faculty members to assess its validity and readability before pretesting among 10 randomly selected consultant physicians for relevance, clarity, and acceptability. Refinements were made as required to facilitate better comprehension and to organize the questions before the final survey was distributed to the research participants. The self-administered questionnaire consisted of socio-demographic questions; questions related to general information about COVID 19; questions related to Pathogenesis & Screening Measures, Management Protocol for COVID-19 and Infection Prevention Measures. Scores of each respondent were calculated by summing up the correct responses.

Upon clicking on the link, the 1st page assured the confidentiality of data, informed the participants of the study objectives, and stated that the participation was purely voluntary. The participant's consent to participate in the study was implied when they clicked on the ‘next’ button to answer the questionnaire, and they had complete freedom either to decline or answer the questionnaire. The study included health care professionals (nurses, physicians, dental surgeons, and allied health professionals) and medical students working /or studying at Chitwan Medical College. Health care professionals and medical students outside Chitwan Medical College were excluded from the study due to various constraints.

The sample size was calculated using the formula,

$$\begin{array}{ll} \text{n} & \text{= }{\text{Z}}^{\text{2}}\times (\text{p}\times \text{q)}/{\text{e}}^{\text{2}}\\ & \text{= }1.96^{\text{2}}\times \{0.7\times (1-0.7)\}/\text{0}{\text{.07}}^{\text{2}}\\ & \text{= }0.806/0.0049\\ & =164.4\\ & =165 \end{array}$$

where,

n = required sample size
p = assuming a knowledge adequacy of 70% among health care professionals based on reported studies^6^
q = 1-p
e = margin of error, 7%
Z = 1.96 at 95% Confidence Interval

Adding a non-response rate of 10%, the calculated sample size was 181.

The distribution of responses was presented as frequencies and percentages. Subgroups were classified based on age, gender, and profession (Nurses, Physicians, Dentists, Allied Health Professionals, and Medical Students).

Adequacy of knowledge was categorized with health care professionals scoring more than 75% (i.e. Score > 15), 60-75% (i.e. Score: 12 - 15) and less than 60% (i.e. Score ≤ 11) as having “Good”, “Fair” and “Poor” knowledge, respectively.

Data were tabulated and analyzed using SPSS (IBM, version 21). Standard descriptive statistics were used. Data were expressed as median (range) or frequency (%) as appropriate. Kruskal Wallis Test was used to compare the knowledge scores among different groups of health care professionals across 4 knowledge domains used in the questionnaire.

## RESULTS

**Figure 1.**
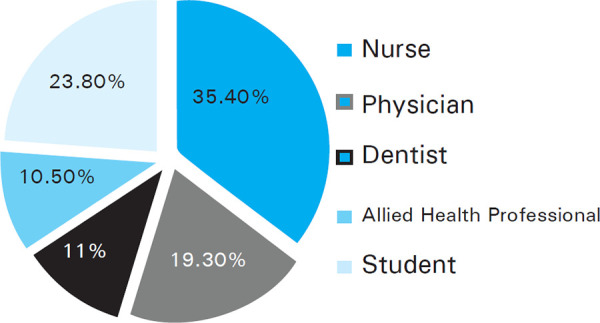
Pie Chart depicting professions of health care respondents.

A total of 181 responders completed the web survey. The median age of the participants was 26 years (Range: 19-60 years). Slightly over one-third of the respondents were Nurses 64 (35.4%) (Figure 1).

The incubation period of COVID-19 was correctly answered as 2-14 days by 153 (84.5%) respondents. Regarding the working case definition for COVID-19 for Nepal, a total of 155 (85.6%) of the respondents correctly identified the Epidemiology and Disease Control Division (EDCD) recommended working case definition of COVID-19 for Nepal. Regarding the stages of the spread of COVID-19, 147 respondents (81.2%) correctly answered that the usual stage of the spread of COVID-19 is - “Imported Cases-Local Transmission-Community Transmission-Epidemic”. A total of 20 (11%) respondents correctly responded that most of the COVID-19 patients present with mild symptoms. Regarding social distancing, a total of 75 (41.4%) respondents correctly answered that social distancing should be at least one meter, as recommended by WHO. A total of 166 (91.7%) respondents correctly answered that as recommended by CDC, the duration of handwashing is at least 20 seconds. Regarding respiratory etiquette, 106 (58.6%) of the respondents correctly answered that respiratory etiquette is understood as coughing or sneezing into a flexed elbow; covering the mouth and nose with a tissue when coughing or sneezing and throwing used tissues in a bin with a lid cover. Regarding PPE Kit contents, a total of 162 (89.5%) of the respondents correctly answered that the N95 mask, cap, goggles or visor, and disposable gown constitute PPE. Regarding isolation, a total of 106 (58.6%) respondents correctly answered that “isolation” separates sick people with a contagious disease from people who are not sick; it is reserved for those who are already sick; it keeps infected people away from healthy people to prevent the sickness from spreading (Table 1).

**Table 1:** Correct responses on various knowledge domains of COVID-19.

KNOWLEDGE DOMAIN: GENERAL INFORMATION ON COVID-19		CORRECT RESPONSE n (%)
	Q. 1. Which virus is responsible for COVID-19?	164 (90.6)
	Q.2. What type of virus is Coronavirus?	147 (81.2)
	Q.3. What is the incubation period of Coronavirus?	153 (84.5)
	Q.4. What is the EDCD recommended working case definition of COVID-19 for Nepal	155 (85.6)
**KNOWLEDGE DOMAIN: PATHOGENESIS & SCREENING OF COVID-19**		
	Q.5. What are the different stages of the spread of the COVID-19	147 (81.2)
	Q.6. What are the Clinical Features of COVID-19	20 (11)
	Q.7. What are the criteria for screening & exclusion of COVID-19	166 (91.7)
**KNOWLEDGE DOMAIN: MANAGEMENT PROTOCOL OF COVID-19**		
	Q.8. What is the correct response to the Case Vignette for assessment of the severity of COVID-19	91 (50.3)
	Q.9. What is the lower limit of oxygen saturation in COVID-19 patient to start Oxygen supplement?	140 (77.3)
	Q.10. When is intubation and mechanical ventilation indicated in COVID-19 patients?	148 (81.8)
	Q.11. What is the inflation pressure for ET Tube to be maintained at all times?	76 (42)
	Q.12. Which one of the following methods is recommended to prevent aerosol generation during patient care?	74 (40.9)
**KNOWLEDGE DOMAIN: INFECTION PREVENTION MEASURES OF COVID-19**		
	Q.13. What is the minimum distance to be maintained in social distancing?	75 (41.4)
	Q.14. What is “isolation”?	106 (58.6)
	Q.15. If you are transferring the suspected or confirmed COVID 19 patient within same hospital what measures are to be followed?	152 (84)
	Q.16. How long should you wash hands to prevent COVID-19?	166 (91.7)
	Q.17. What is Respiratory Etiquette?	106 (58.6)
	Q.18. What does the PPE kit consist of if you are performing or assisting in aerosol generating procedures?	162 (89.5)
	Q.19. What is the recommended strength of chlorine while disinfecting the environment in fever clinic?	68 (37.6)
	Q.20. Safe breast feeding practices by a mother with COVID-19	97 (53.6)

The respondents scored the highest for the question about the duration of hand washing which was correctly answered as “at least 20 seconds” by 166 (91.7%) of the respondents. The respondents scored the lowest for the question about clinical features of COVID-19 which was correctly answered as “most of the patients have mild symptoms” by only 20 (11%) of the respondents.

Overall, a total of 35 (19.3%) respondents were found to have “Good” knowledge; 105 (58%) respondents have “Fair” knowledge and 41 (22.7%) respondents have “Poor” knowledge regarding various aspects of COVID-19.

There was no significant difference among the various health professional groups in their knowledge scores under the four knowledge domains (Table 2).

**Table 2:** Median Score of Health Care Professionals across Various Knowledge Domains.

	General Information on COVID-19	Pathogenesis & Screening of COVID-19	Management of COVID-19	Infection Prevention Measures for COVID-19	Total
Nurse(n = 64)	3 (0-4)	2 (0-3)	3 (0-5)	5.5 (2-8)	14 (4-18)
Physician (n = 35)	4 (2-4)	2 (0-3)	3 (1-5)	5 (3-8)	14 (9-17)
Dentist (n = 20)	4 (1-4)	2 (1-3)	3 (1-5)	5 (3-7)	13.5 (917)
Allied Health Professional (n = 19)	4 (2-4)	2 (1-3)	3 (1-5)	5 (3-7)	14 (11-17)
Student (n = 43)	4 (2-4)	2 (1-3)	3 (0-5)	5 (2-7)	13 (8-17)

## DISCUSSION

Health care professionals, who are the mainstay of health care workers in medical colleges and teaching hospitals have always been at risk of infectious diseases, and the explosive spread of COVID-19 has increased their risk many folds.

Knowledge is a prerequisite for establishing beliefs on prevention, for the formation of positive attitude and promotion of positive behaviour change. The effectiveness of coping strategies and behaviours is largely affected by an individual's cognition and attitude towards diseases.^7^ Therefore the analysis of knowledge of health care professionals regarding COVID-19 could act as a reference for prevention of the epidemic of COVID-19.^8^

In this study, more than two-thirds of the respondents had adequate (“good” knowledge and “fair” knowledge category) knowledge regarding various aspects of COVID-19.

In this study, 84.5% of the respondents knew about the incubation period of COVID-19, which is 2-14 days. This finding is similar to the finding in a study by Bhagavathula et al. in UAE,^9^ Khader et al. in Jordan,^10^ and Taghrir et al. in Iran.^11^ It is essential to know the right incubation period because it helps in determining the safe period to treat suspected patients.^12^

Most of the respondents (85.6%) in this study were aware of the Epidemiology and Disease Control Division (EDCD) recommended working case definition of COVID-19 for Nepal, which includes - Acute Respiratory Illness; travel history, or residence in a country, area or territory that has reported local transmission of COVID-19 during the 14 days before symptom onset; has been in close contact with probable/confirmed case of COVID-19.^13^ Being well informed of the working case definition of a country is of paramount importance and goes a long way in case identification, contact tracing, isolation and quarantine as well as timely management of COVID-19 patients.

The World Health Organization recommends a social distancing of at least one metre between oneself and others.^14^ In this study, only 40.9% of the respondents correctly mentioned one metre of social distancing. Social distancing is designed to reduce interactions between people in a community, in which individuals may be infectious but have not yet been identified hence not isolated.^15^ As diseases transmitted by respiratory droplets require certain proximity of people, the social distancing of persons is needed to reduce transmission. This finding of the study needs urgent attention because health care professionals are the front liners who play a vital role in disseminating correct and factual messages to the public and also act as a role model for the public. Hence, it is imperative to build upon the correct knowledge base of health care professionals regarding COVID-19.

It is evident from the COVID-19 pandemic that effectively applied hand hygiene is a vital intervention that can be used to prevent the spread of disease.^16^ The Centre for Disease Control (CDC) recommends at least twenty seconds of handwashing to prevent COVID-19.^17^ In this study, 91.7 % of the respondents were aware of the handwashing duration recommended by the CDC. The importance of handwashing, the correct steps of handwashing and the correct duration of handwashing have been well reinforced by various media sources in Nepal and this could be a prominent reason why knowledge on the duration of hand hygiene was correctly known to a majority of respondents.

As per WHO, respiratory etiquette is understood as coughing or sneezing into a flexed elbow; covering the mouth and nose with a tissue when coughing or sneezing and throwing used tissues in a bin with a lid cover. ^14^ In this study, only 58.6% of the health care professionals were aware of the exact concept of respiratory etiquette. This finding again calls for a reinforcement of infection prevention measures to the health care professionals.

Awareness of the use of personal protective equipment (PPE) for suspected/confirmed COVID-19 cases were high among all groups of healthcare professionals; almost 90% of the respondents could identify the PPE kit needed while performing/assisting in aerosol-generating procedures.

In a study in Mumbai, India by Modi et al. 79% of the respondents were aware of the various personal protective equipment (PPE) recommended for use in suspected COVID-19 patients in a healthcare setting.^6^

The CDC has provided Interim Infection Prevention and Control Recommendations for Patients with suspected or confirmed coronavirus disease 2019 (COVID-19) in Healthcare Settings for PPE.^18^ A Facemask/N95 respirator should be worn when entering into the patient room. The N95 respirator is preferred over face mask while performing aerosol-generating procedures. A clean gown with goggles/visor and clean non-sterile gloves are recommended upon entry to the patient room area. In case of shortage, gowns should be prioritized for aerosol-generating procedures.^18^

WHO recommends that mothers with confirmed or suspected COVID-19 can breastfeed their babies provided they follow respiratory etiquette and hand hygiene.^19^ However, in this study, only 53.6% of the respondents were aware of this fact. This finding is of concern given the fact that optimal breastfeeding recommended by WHO emphasizes on breastfeeding within the first hour of birth, exclusive breastfeeding for the first six months of life and from the age of six months, children should begin eating safe and adequate complementary foods while continuing to breastfeed for up to 2 years and beyond.^20^ It is therefore imperative that all health care professionals are well aware of the concept of optimal breastfeeding, more so in the context of COVID-19 and disseminate this message adequately.^20^

Overall, the respondents had good knowledge of the general information; pathogenesis and screening, as well as infection prevention measures regarding COVID-19, with no differences, observed between the various groups of health care professionals. However, the respondents scored the lowest in the domain related to the management of COVID-19. This could be ascribed to questions which were tad bit technical in this domain and also because confirmed cases of COVID-19 have been few and far between in Chitwan, so far.

The analysis of knowledge of health care professionals regarding COVID-19 could act as a reference for the prevention and better management of COVID-19. This study shows that there is a need to implement periodic educational interventions and training programs on infection control practices for COVID-19 across all healthcare professions.

This is a cross-sectional study conducted online among health care professionals during a time (April 22, 2020, to April 28, 2020) when an alarming number of cases were being reported globally and this might limit generalizations. The data presented in this study are self-reported and partly dependent on the respondent's honesty and recall ability. Thus, they may be subject to recall bias. As the response rate was only 25%, the results of this study may limit generalizations.

## CONCLUSIONS

Health care professionals from Chitwan Medical College showed adequate awareness of COVID-19. Health care professionals have a decisive role in infection control, case identification, isolation, contact tracing, quarantine and management of COVID-19 patients. Knowledge is a prerequisite for establishing beliefs on prevention, for the formation of positive attitude and promotion of positive behaviour change. The effectiveness of coping strategies and behaviours is largely affected by an individual's cognition and attitude towards diseases. Therefore the analysis of knowledge of health care professionals regarding COVID-19 could act as a reference for the prevention and better management of COVID-19. This study shows that there is a need to implement periodic educational interventions and training programs on infection control practices for COVID-19 across all healthcare professions. Health Care Professionals are advised to follow the CDC and WHO guidelines in their health care settings to adequately brace for the COVID-19 pandemic.

## Conflict of Interest

**None.**
